# Supplementary material for: Length‐independent telomere damage drives post‐mitotic cardiomyocyte senescence
Source: EMBO J. 2019 Feb 8;38(5):e100492. doi: 10.15252/embj.2018100492 (PMC6396144; doi:10.15252/embj.2018100492)
Supplement: Supplementary file 1 — Appendix [file EMBJ-38-e100492-s001.pdf]

Table of contents:

7 Appendix Supplementary Figures;

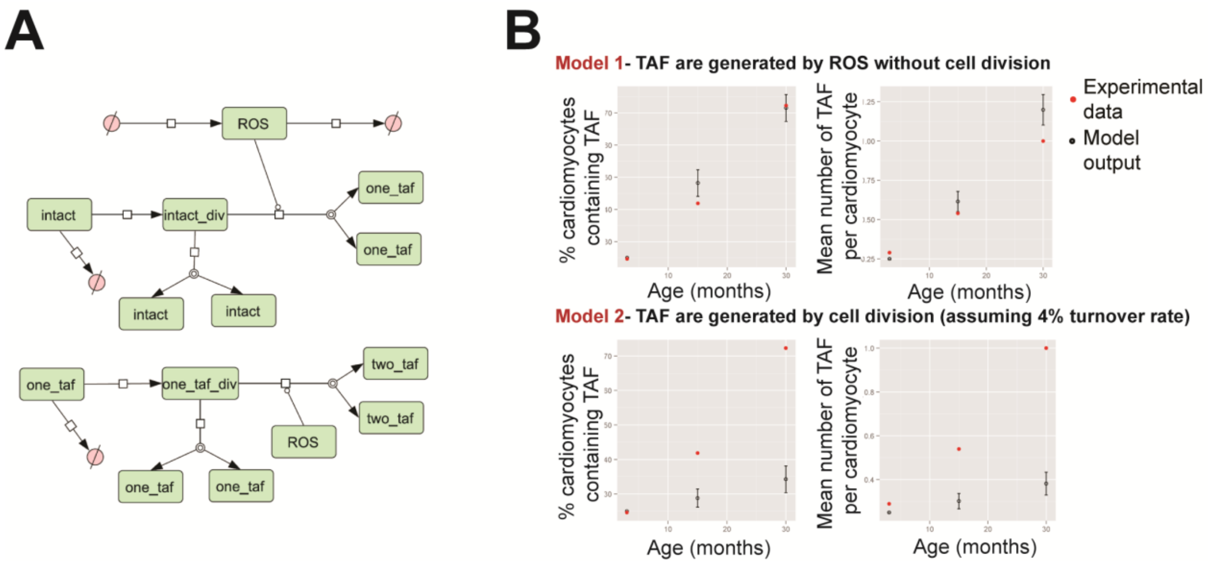

Appendix Figure S1 Stochastic mathematical models representing a cardiomyocyte population throughout 27 months of a mouse's lifespan A) Network diagram of **Model 2**,- TAF are generated by cell division. Reactions involving turnover of cells with two, three or four TAF are similar to those for cells with one TAF and are omitted for clarity. Network diagram created in CellDesigner using Systems Biology Graphical Notation. **B)** Middle and bottom panel: model output showing mean values of 100 stochastic simulations, (error bars show ± one s.d. from the mean).

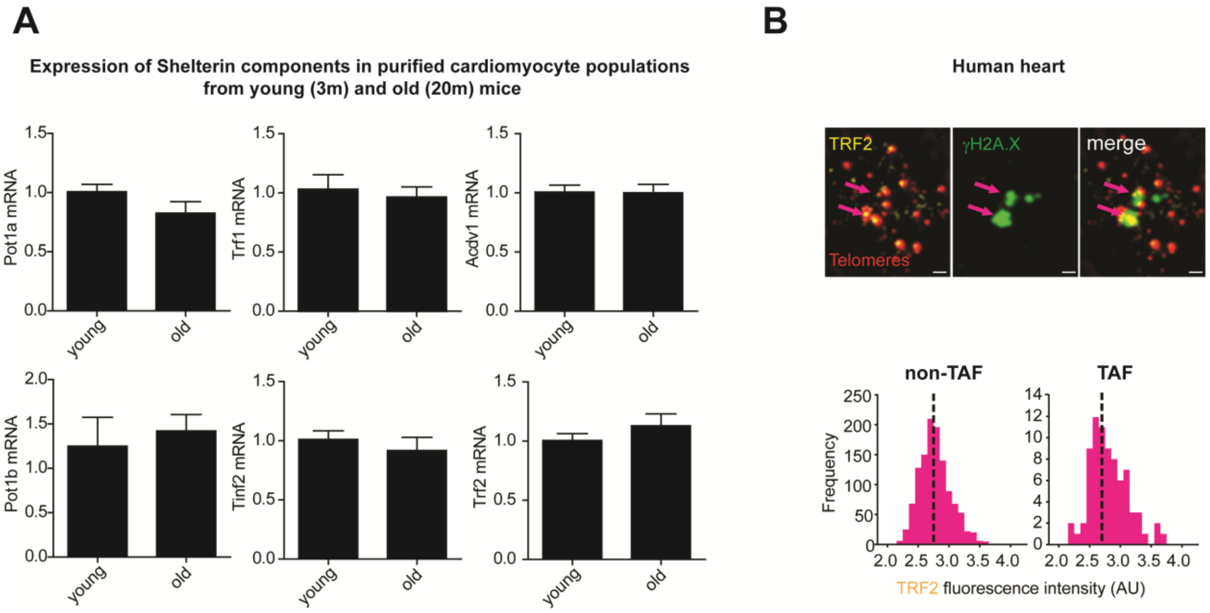

Appendix Figure S2 Expression of shelterin components does not change with age in cardiomyocytes.

A) mRNA expression of shelterin components: Pot1a, Pot1b, Trf1, Trf2, Tinkf2, and Acdv1 in purified cardiomyocyte populations from young (3m) and old (20m) wild-type mice. Data are mean ± S.E.M of n=5 mice per group. Two-tailed t-test shows no significant difference in expression (P>0.05). **B)** Representative image of TRF2 immuno-FISH in PCM1-positive human cardiomyocytes (blue – DAPI; yellow – TRF2; red – telo-FISH; green – γH2A.X). Images are z projections of 4.5µM stacks taken with 100X objective. Scale bar represents 1µm. (below) Histograms displaying TRF2 fluorescence intensity for TRF2 foci co-localising with telomeres also co-localising with γH2A.X (right) or telomeres not co-localising with γH2A.X (left) in human cardiomyocytes. Red dotted lines represent median intensity. Mann-Whitney tests show no significant difference in TRF2 intensity between TRF2 abundance at TAF and non-TAF (P>0.05).

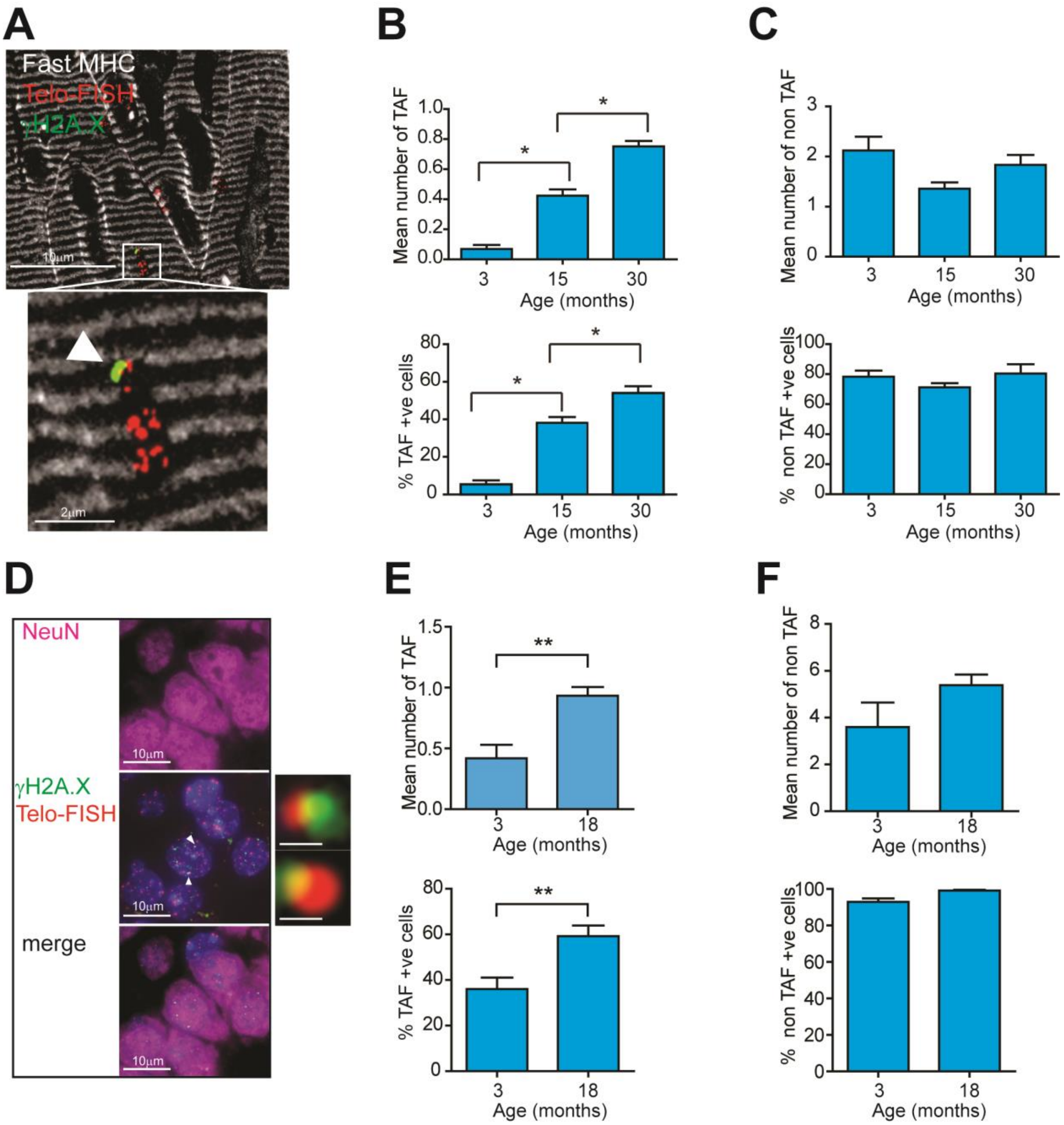

Appendix Figure S3 Telomere-dysfunction increases with age in Post-mitotic cells such as muscle fibres and neurones

A) Representative image of Immuno-FISH for γH2A.X, telomeres and fast MHC (marker of type 2 muscle fibres). White arrow indicates co-localisation between γH2A.X and telomeres; **B)** Mean number of TAF (top) and % of myocytes positive for TAF (bottom); **C)** Mean number of non-TAF (top) and % of myocytes containing non-TAF (bottom) in 3,15, and 30 month old mice. Data are mean ± SEM of n=4 per age group. More than 70 myocytes were quantified per age group. Statistical analysis performed using One Way ANOVA; * P<0.05; **D)** representative image of Immuno-FISH for γH2A.X, telomeres and NeuN (neuronal marker) in mouse hippocampus. White arrows indicate co-localisation between γH2A.X and telomeres. Scale bars as indicated or 500nm in examples of individual TAF. **E)** Mean number of TAF (top) and % of neurons positive for TAF (bottom); **F)** Mean number of non-TAF (top) and % of neurons containing non-TAF (bottom) in 3 and 18 month old mice. Data are mean ± SEM of n=5-7 per age group. More than 100 neurons were quantified per age group. Statistical analysis performed using two-tailed t-test; * P<0.05.

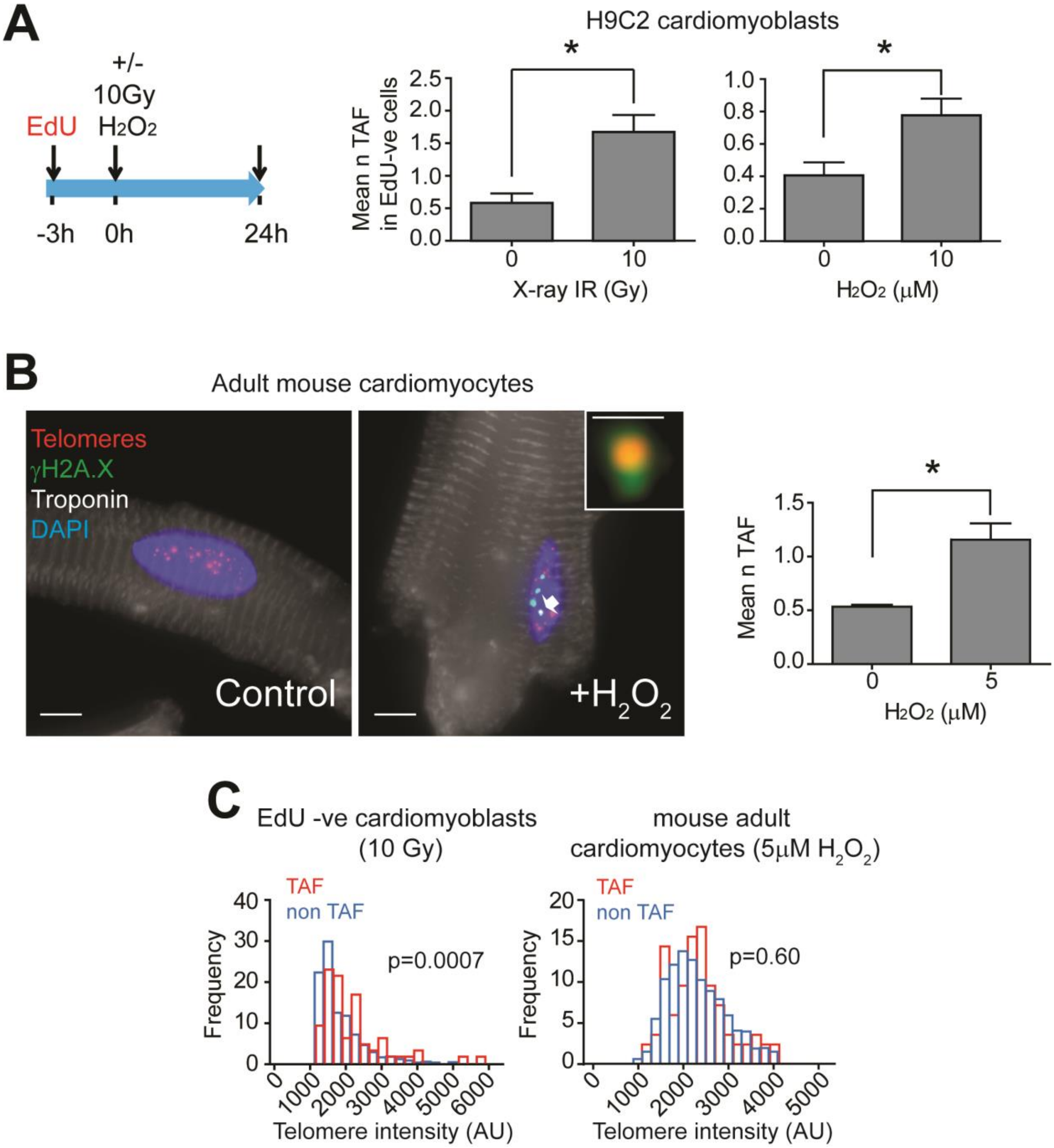

Appendix Figure S4 Telomere damage occurs irrespectively of cell division

A) Schematic illustration depicting H9C2 cells were incubated in 10μM EdU in normal growth medium for 3 hours, followed by either 10Gy X-irradiation or incubation with 10μM H_2O_2 and cultured for a further 24 hours in the presence of 10μM EdU in normal growth medium. Mean number of TAF in EdU positive cells following X-irradiation (left graph) or H_2O_2 (right graph). Data are mean ± SEM of *n*=3 independent experiments. 50 cells were analysed per experiment. Statistical analysis performed using two-tailed t test; * P<0.05. **B)** (left) Representative micrographs of Immuno-FISH for γH2A.X, telomeres and Troponin C in control and H_2O_2 treated adult mice cardiomyocytes. Arrow indicates co-localisation between telomeres and γH2A.X. In main image scale bar corresponds to 10μm, in individual TAF example scale bar represents 500nm. (right) Mean number of TAF in isolated mouse adult cardiomyocytes 24hr after treatment with 5μM H_2O_2. Data are mean ± SEM of adult cardiomyocytes isolated from 3 month-old C57BL/6 wild-type mice. Statistical analysis performed using two-tailed t test; * P<0.05. **C)** Histograms representing Q-FISH analysis

comparing individual telomere intensities of telomeres either co-localising (TAF) or not co-localising (non-TAF) with γH2AX DDR foci in 10Gy irradiated EdU-negative H9C2 cardiomyoblasts (left graph) or 5μM H_2O_2 treated mouse adult cardiomyocytes from 3 month-old C57BL/6 wild-type mice (n=3 mice). More than 100 cells were analysed per experiment. Statistical analysis performed using Mann Whitney test.

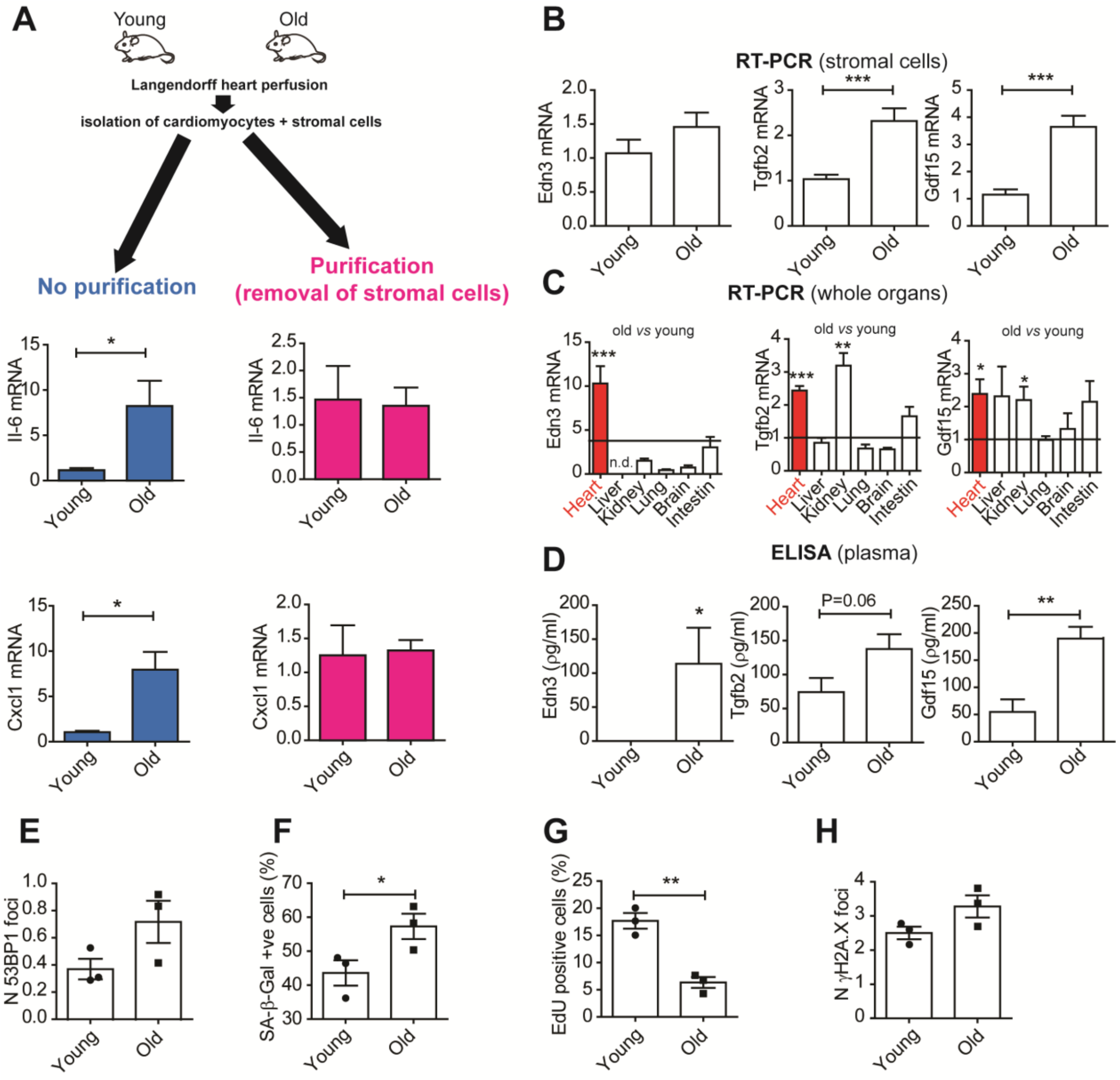

Appendix Figure S5 Senescent cardiomyocytes have a non-canonical SASP phenotype A) Stromal cells isolated as part of the *Langendorff* heart perfusion contribute to the previously reported increased SASP in aged cardiomyocytes: (left) Using the traditional cardiomyocyte isolation method, significant increases in mRNA expression of SASP components Il-6 and Cxcl1 are observed with age; (right) Following removal of stromal cells, no significant differences could be found. Data are mean ±S.E.M of n=4-6 mice per group; **B)** Stromal cells (no cardiomyocytes) show age-dependent increased expression of Tgfb2 and Gdf15 but not Edn3. Data are mean ±S.E.M of n=8 mice per group. **C)** Only Edn3 shows an age-dependent increase in expression in the heart. Tgfb2 and GDF15 increase significantly both in heart and kidney. Data are mean ±S.E.M of n=4-7 mice per group. **D)** Edn3, Tgfb2, and Gdf15 increase with age at the protein level in plasma. Mouse adult ear fibroblasts were treated with conditioned media from young (3m) and old (20m) cardiomyocytes for 3 days and analysed for: **E**) N of 53BP1, **F**) SA-β-Gal and **G**) EdU. **H)** Neonatal cardiac fibroblasts were treated with conditioned media from young (3m) and old (20m) cardiomyocytes for 96h and analysed for γH2A.X foci. Data are mean ±S.E.M of n=3 mice per group.

Statistical analysis: for multiple comparisons One-way ANOVA was used, otherwise two-tailed t-test. ***P<0.0001; **P<0.01; * P<0.05.

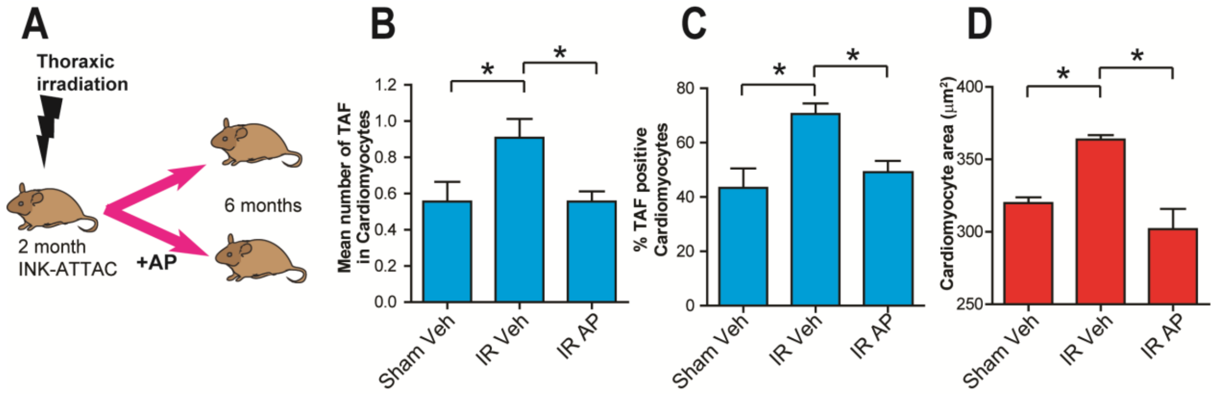

Appendix Figure S6 Genetic clearance of p16^{Ink4a} positive cells rescues telomere dysfunction and cardiac hypertrophy after thoracic irradiation. **A)** Schematic depicting experimental design for figures e-g: 2 month old INK-ATTAC mice underwent thoracic X-irradiation, were treated with AP20187 (or Vehicle) and then sacrificed at 6 months of age; **B)** Mean number of TAF; **C)** percentage of TAF-positive cardiomyocytes and **D)** mean cardiomyocyte area μm^2 in sham irradiated, and irradiated mice with or without AP treatment. Data are mean ± SEM of *n*=6-10 per age group. More than 100 cardiomyocytes were quantified per group. Asterisks denote $P < 0.05$ using one-way ANOVA;

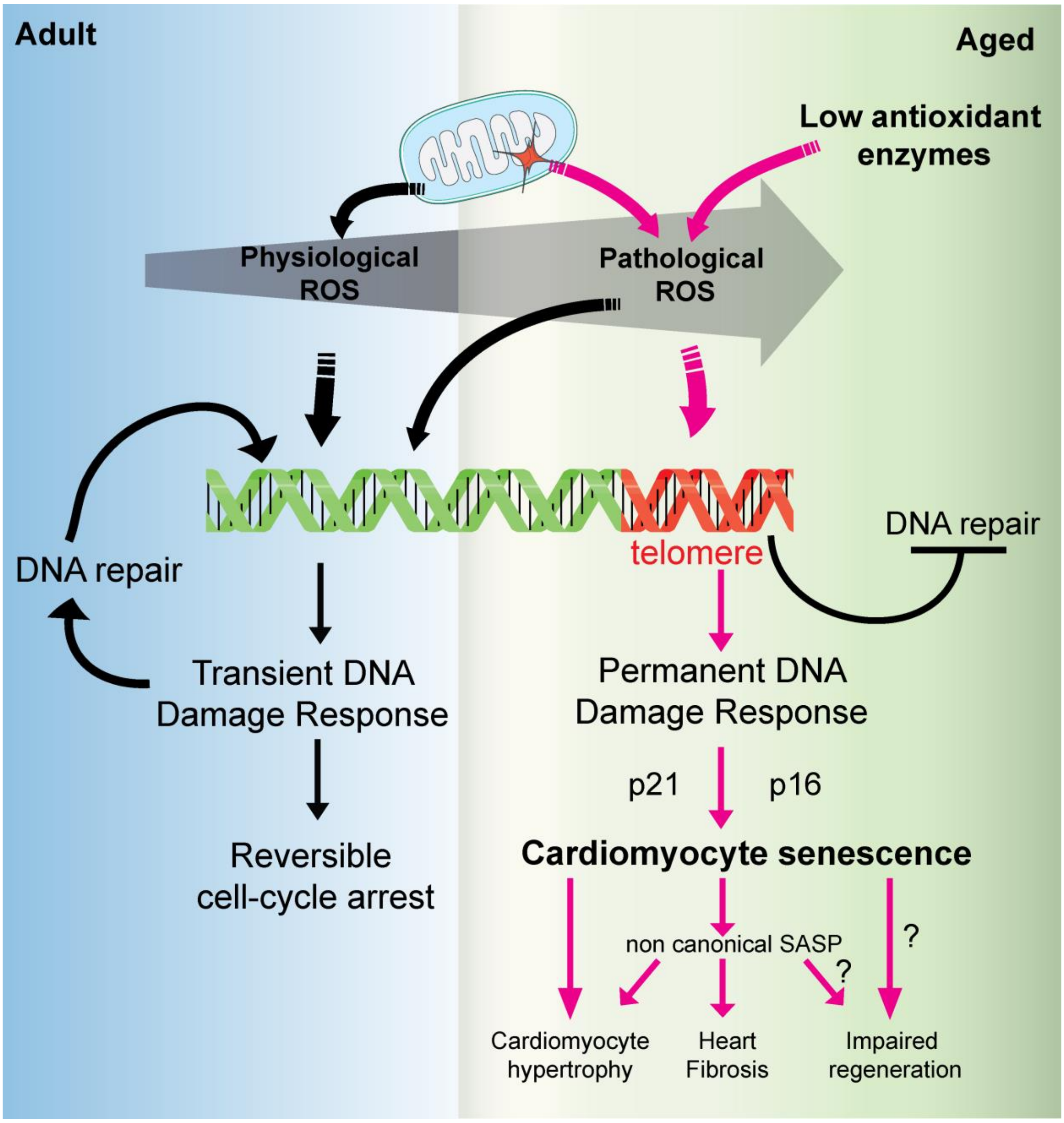

Appendix Figure S7 Model to explain cardiomyocyte senescence. In young adult mice, a continuous cycle of “physiological” low level ROS maintains a transient DDR which is a contributor to the low myocardial turnover. During ageing, mitochondrial dysfunction and low expression of antioxidant enzymes, induces “pathological” high ROS which randomly induces telomere-dysfunction that results in a permanent DNA Damage Response sufficient to robustly activate p21 and p16 senescence pathways. Cardiomyocyte senescence (CS) is a driver of myocardial hypertrophy and is characterised by a distinct SASP which contributes to fibrosis and hypertrophy. CS may contribute to impaired regeneration; however the latter hypothesis remains to be tested experimentally.
