# Supplementary material for: Length‐independent telomere damage drives post‐mitotic cardiomyocyte senescence
Source: EMBO J. 2019 Feb 8;38(5):e100492. doi: 10.15252/embj.2018100492 (PMC6396144; doi:10.15252/embj.2018100492)
Supplement: Supplementary file 2 — Expanded View Figures PDF [file EMBJ-38-e100492-s002.pdf]

Expanded View Figures

Figure EV1. Aged cardiomyocytes in both humans and mice are characterised by dysfunctional telomeres.

A Representative images of γH2A.X immuno-FISH in PCM1-positive human cardiomyocytes (blue—DAPI; yellow—PCM1; red—telo-FISH; green—γH2A.X). Images are z-projections of 4.5 μm stacks taken with 100× objective. Right panels show co-localisation between telomeres and γH2AX, with taken from single z-planes where co-localisation was found. Graphs showing mean number of TAF (left) and mean percentage of TAF-positive nuclei (right) in PCM1-positive human cardiomyocytes from 46-year-old to 65-year-old and 74-year-old to 82-year-old human heart tissue. Data are represented as the mean for individual subjects, with the horizontal line representing group mean. *$P < 0.05$ statistical significance using two-tailed *t*-test. Scale bars as indicated.

B Quantification of 53BP1 Immuno-FISH in 4-month-old and 24-month-old mice. Graphs representing mean number of TAF and % of cardiomyocytes positive for TAF are in black and graphs representing mean number of 53BP1 and % of cardiomyocytes positive for 53BP1 are in orange; $n = 3$ mice per age group; > 100 cardiomyocytes were quantified. Asterisk denotes a statistical significance at $P < 0.01$ using two-tailed *t*-test.

C Above: Representative images of PCM1 and α-actinin, PCM1 and WGA and γH2A.X, PCM1 immuno-FISH in 30-month-old mouse cardiac tissue. White arrows identify PCM1-expressing CM nuclei. Below: Graph representing mean ± SEM number of TAF in PCM1-positive versus PCM1-negative cardiac cells; $n = 3$ mice; > 100 cardiomyocytes were quantified. Asterisk denotes a statistical significance at $P < 0.01$ using two-tailed *t*-test. Scale bars represent 20 μm.

D Representative images of conventional confocal (green) versus STED microscopy (red) for detection of telomeres by Q-FISH. Blue arrows identify telomeres shown in higher magnification in right 2-panels. Note that STED microscopy is capable of discerning telomere clusters which would otherwise be detected by confocal microscopy as a single signal. (left) Main image scale bar represents 1 μm. (Right) Higher magnification images scale bars represent 500 μm.

E Comparison between average number of telomere FISH signals detectable per cell (left graph) and mean telomere volume (right graph) in mouse cardiomyocytes, detected by either standard confocal or STED microscopy. Data are represented as the mean ± SEM for each measurement, with the horizontal line representing the group mean.

F Representative image of immuno-FISH using STED microscopy for γH2A.X and telomeres in cardiomyocytes from a 30-month-old mouse. Graph on the right side shows two telomere FISH signals of similar intensities (one showing co-localisation with γH2A.X and the other not). Scale bar represents 1 μm.

G Histograms representing Q-FISH analysis by 3D STED microscopy comparing individual telomere length either co-localising (TAF) or not co-localising (non-TAF) with γH2AX foci in mouse cardiomyocytes from $n = 3$ mice (aged 30 months of age). 300 cardiomyocytes (detected by troponin-C and WGA) were analysed per mouse. Mann–Whitney test reveals no statistical significance between TAF and non-TAF $P = 0.13$.

H Histograms displaying telomere intensity for telomeres co-localising (bottom) or not co-localising (top) with γH2A.X DDR foci for PCM1-positive cardiomyocytes obtained from 46-year-old to 65-year-old (left) and 74-year-old to 82-year-old subjects. Dotted lines represent median intensity. Mann–Whitney test show no significant difference in telomere intensity between TAF and non-TAF in either 46-year-old to 65-year-old or 74-year-old to 82-year-old subjects ($P > 0.05$). More than 100 cardiomyocytes were quantified per subject.

I Graph showing values for telomere FISH intensities either co-localising (TAF) or not co-localising (non-TAF) with γH2AX foci in 22 individual cardiomyocytes chosen randomly. Purple arrow indicates one telomere FISH signal, which is lower in intensity than the median. Green horizontal lines denote median fluorescence intensity.

 © 2019 The Authors

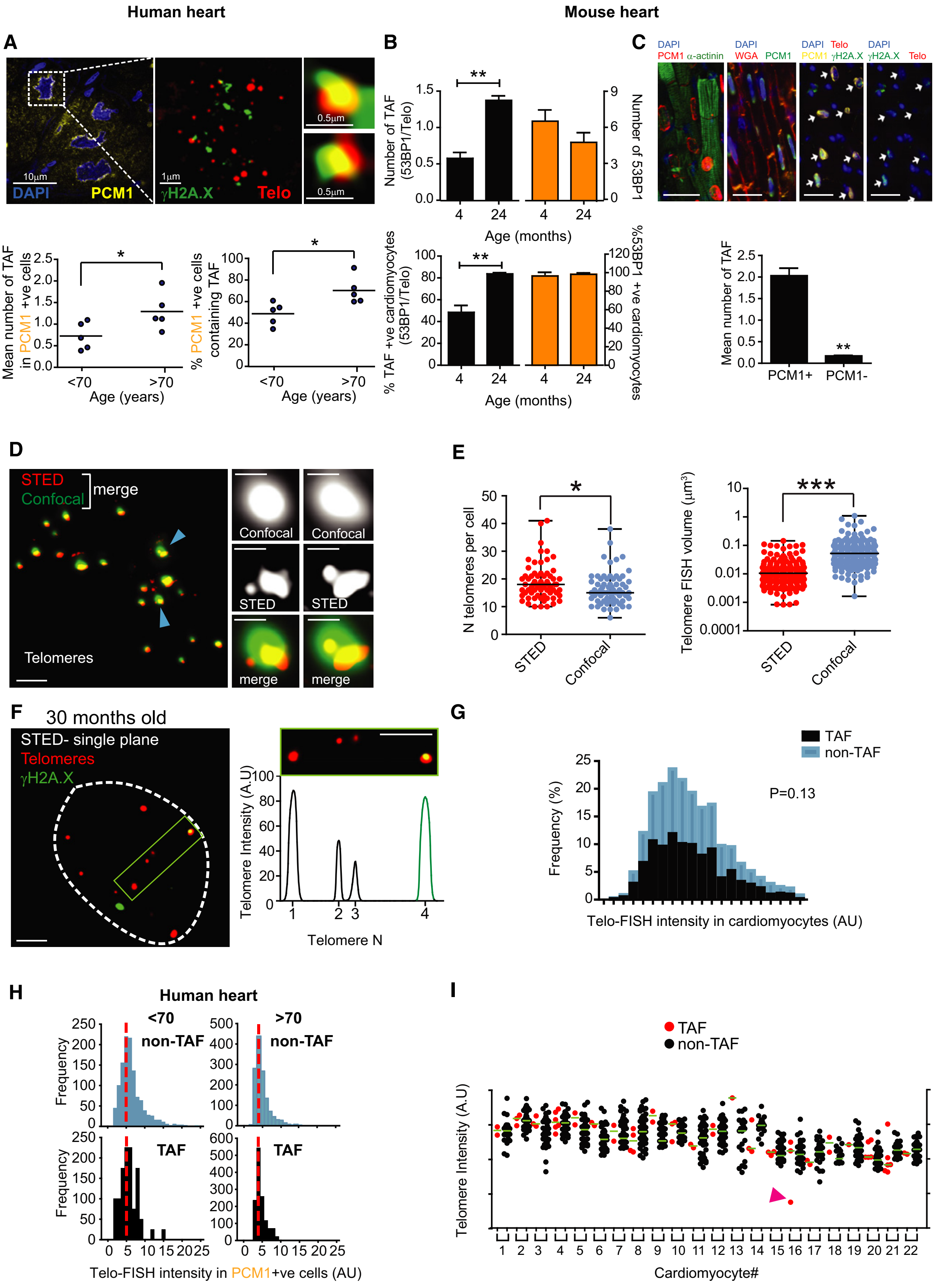
Human heart
Mouse heart
A
B
C
10μm
1μm
0.5μm
DAPI
PCM1
γH2A.X
Telo
Number of TAF (53BP1/Telo)
Number of 53BP1
Age (months)
WGA
PCM1 α-actinin
Mean number of TAF in PCM1 +ve cells
% PCM1 +ve cells containing TAF
Age (years)
<70
>70
% TAF +ve cardiomyocytes (53BP1/Telo)
%53BP1 +ve cardiomyocytes
Mean number of TAF
PCM1+
PCM1-
D
STED
Confocal
merge
Telomeres
E
N telomeres per cell
Telomere FISH volume (μm³)
F
30 months old
STED- single plane
Telomeres
γH2A.X
Telomere Intensity (A.U)
Telomere N
G
TAF
non-TAF
P=0.13
Frequency (%)
Telo-FISH intensity in cardiomyocytes (AU)
H
Human heart
<70
>70
non-TAF
TAF
Frequency
Telo-FISH intensity in PCM1+ve cells (AU)
I
TAF
non-TAF
Telomere Intensity (A.U)
Cardiomyocyte#

Figure EV1.

Figure EV2. TRF1-FokI fusion protein induces telomere-specific double-strand breaks, senescence and hypertrophy in H9C2 cardiomyoblasts.

- A Representative images of H9C2 cardiomyoblasts 4 days following transfection with a FLAG-tagged TRF1-FokI-D450A (top row) or TRF1-FokI (middle and bottom row) fusion protein (cell treatments the same for all subsequent panels in figure; purple—FLAG; red—telo-FISH; green—γH2A.X/53BP1). Images are z-projections of 0.1 μm stacks taken with 100× objective. White arrows indicate co-localisation between telomeres and γH2A.X/53BP1, with co-localising foci amplified in the right panels (taken from single *z*-planes where co-localisation was found). Scale bars main image represent 2.5 μm. Scale bars single-plane images 500 nm.
- B Percentage of γH2A.X (left) or 53BP1 (right) foci co-localising with telomeres. Data are mean ± SEM of $n = 3$ independent experiments. More than 50 cells were analysed per condition. Statistical analysis was performed by two-tailed *t*-test; $^*P < 0.05$.
- C Histograms displaying telomere intensity for telomeres co-localising (bottom) or not co-localising (top) with γH2AX (left) or 53BP1 (right) DDR foci. Red dotted lines represent median. Statistical analysis performed using two-tailed *t*-test. More than 50 cells were analysed per condition. Mann–Whitney tests show no significant difference in telomere intensity between TAF and non-TAF, with either γH2A.X (left) or 53BP1 (right) DDR foci.
- D Representative images of detection of senescent markers (purple—FLAG; red—Ki-67; light blue—DAPI; darker cytoplasmic blue—SA-β-Gal). Scale bar represents 10 μm.
- E Mean percentage of FLAG-positive cells positive for Ki-67, p21, SA-β-Gal activity and cell size. Data are mean ± SEM of $n = 3$ independent experiments. More than 100 cells were quantified per condition. For SA-β-Gal, data are presented as mean of > 100 cells representative of 1 experiment. Two additional independent experiments confirmed these findings (not shown). Statistical analysis performed using two-tailed *t*-test; $^*P < 0.05$.
- F Scheme depicting experimental setting: AC10 human cardiomyocyte cell line stably expressing an inducible TRF1-FokI was cultured for 2 weeks in mitogen-depleted media which led to terminal differentiation and loss of proliferation. Then, cells were treated for 6 h with doxycycline (DOX), after which DOX was removed and cells were allowed to recover for 36 h.
- G Mean number of TAF following induction of TRF1-FokI and recovery. Data are mean ± SEM of $n = 4$ independent experiments. Statistical analysis performed using one-way ANOVA (Holm–Sidak method); $^*P < 0.05$.
- H % of SA-β-Gal-positive cells expressing TRF1-FokI 36 h after 6 h DOX treatment. Data are mean ± SEM of $n = 4$ independent experiments. Statistical analysis performed using two-tailed *t*-test; $^*P < 0.001$.
- I Forty-eight hours post-transfection, neonatal rat cardiomyocytes were stimulated with 4-OH-T (300 nM) during 6 h for nuclear re-localisation of I-PpoI and DSD induction. Analysis of γH2A.X was performed immediately after treatment and at day 4 post-treatment. Data are mean ± SD from $n = 50$ cells per condition from two independent experiments. Scale bars represent 10 μm.
- J % of SA-β-Gal-positive neonatal cardiomyocytes transfected with inducible I-PpoI at day 4 post-treatment. Data are mean ± SD from $n = 50$ cells per condition from two independent experiments.
- K Analysis of cell size in neonatal cardiomyocytes transfected with inducible I-PpoI at day 4 post-treatment. Data are mean ± SEM from $n = 50$ cells per condition from four independent experiments.

© 2019 The Authors

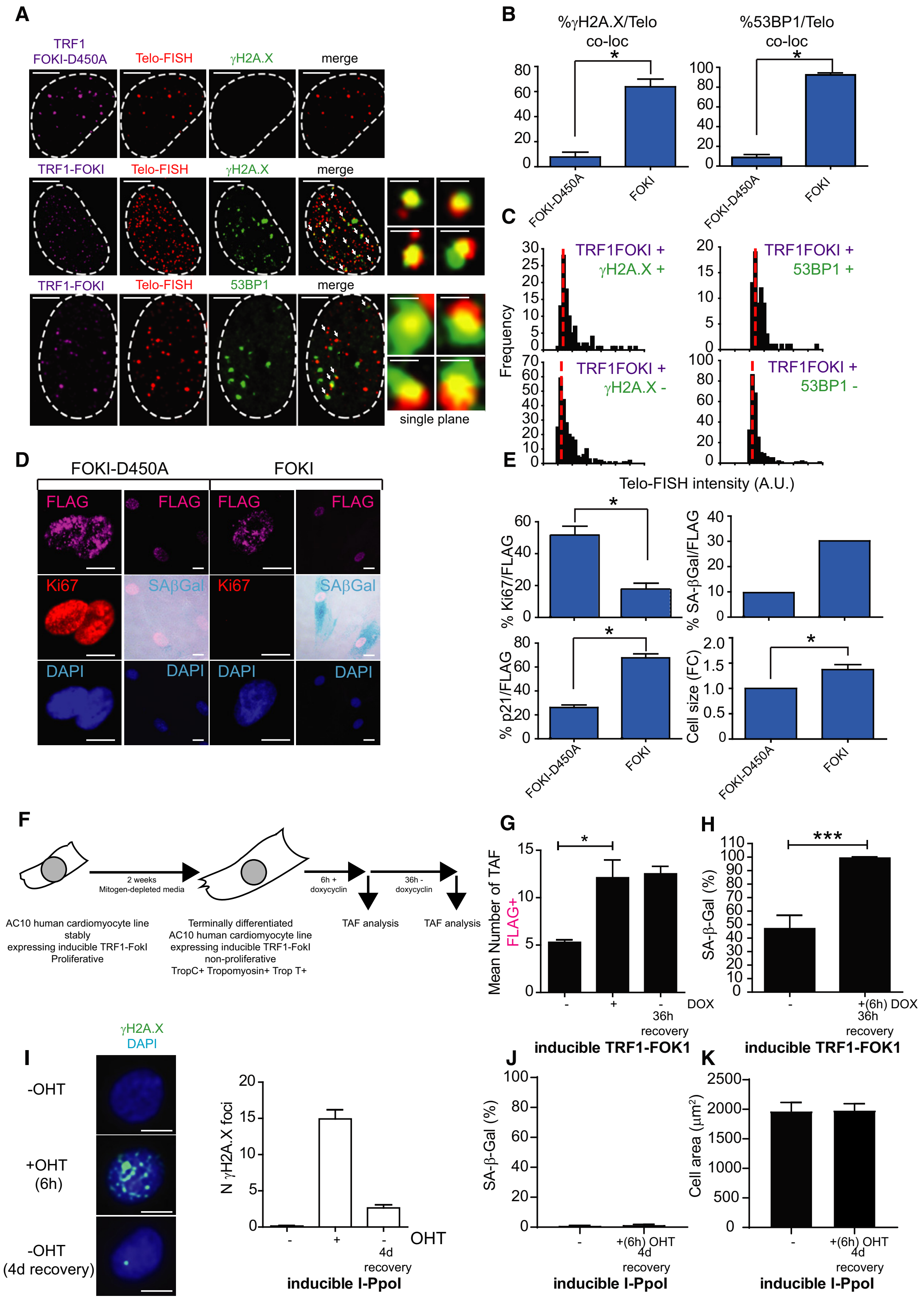
A
TRF1 FOKI-D450A
Telo-FISH
γH2A.X
merge
TRF1-FOKI
53BP1
single plane
B
%γH2A.X/Telo co-loc
%53BP1/Telo co-loc
FOKI-D450A
FOKI
C
Frequency
TRF1FOKI + γH2A.X +
TRF1FOKI + 53BP1 +
TRF1FOKI + γH2A.X -
TRF1FOKI + 53BP1 -
Telo-FISH intensity (A.U.)
D
FOKI-D450A
FOKI
FLAG
Ki67
SAβGal
DAPI
E
% Ki67/FLAG
% SA-βGal/FLAG
% p21/FLAG
Cell size (FC)
F
2 weeks Mitogen-depleted media
6h + doxycyclin
36h - doxycyclin
TAF analysis
AC10 human cardiomyocyte line stably expressing inducible TRF1-FokI Proliferative
Terminally differentiated AC10 human cardiomyocyte line expressing inducible TRF1-FokI non-proliferative TropC+ Tropomyosin+ Trop T+
G
Mean Number of TAF FLAG+
36h recovery
DOX
inducible TRF1-FOK1
H
SA-β-Gal (%)
+(6h) DOX
I
γH2A.X
DAPI
-OHT
+OHT (6h)
-OHT (4d recovery)
N γH2A.X foci
4d recovery
OHT
inducible I-PpoI
J
+(6h) OHT
K
Cell area (μm²)

Figure EV2.

Figure EV3. Aged cardiomyocytes have no alterations in proliferation gene expression but show increased expression of genes associated with hypertrophy.

A Representative images of cardiomyocytes isolations before (left) and after (right) purification to remove SCA1-, CD31-, and CD45-positive cells. Scale bar represents 20 μm.

B Counts of differentially expressed genes calculated using DESeq2 at % 5 FDR.

C Principal component analysis (PCA) of FPKM expression for young (red) and old (blue) cardiomyocytes. Components one and two account for 80.1 and 7.8% of the total variance, respectively.

D Correlation clustered heatmap of a curated list of known proliferation genes in young and old cardiomyocytes. The colour intensity represents column *Z*-score, with red indicating high expression and blue low expression. Note that there is no enrichment for differential expression in this subset of pro-proliferation genes.

E Trace plots and heatmap of the relative expression of Ankrd1, Capn3, Mybcp2, Myot, Myom3 and their associated FDR-corrected *q*-values derived from DESeq2.

F Confirmatory RT–PCRs showing changes in cardiac hypertrophy genes Acta1 and Myh7, but not Anf. Data were normalised to Gapdh. Data are mean ± SEM of n = 4–5 mice per age group. Two-tailed t-test was used. **P < 0.01; *P < 0.05.

© 2019 The Authors

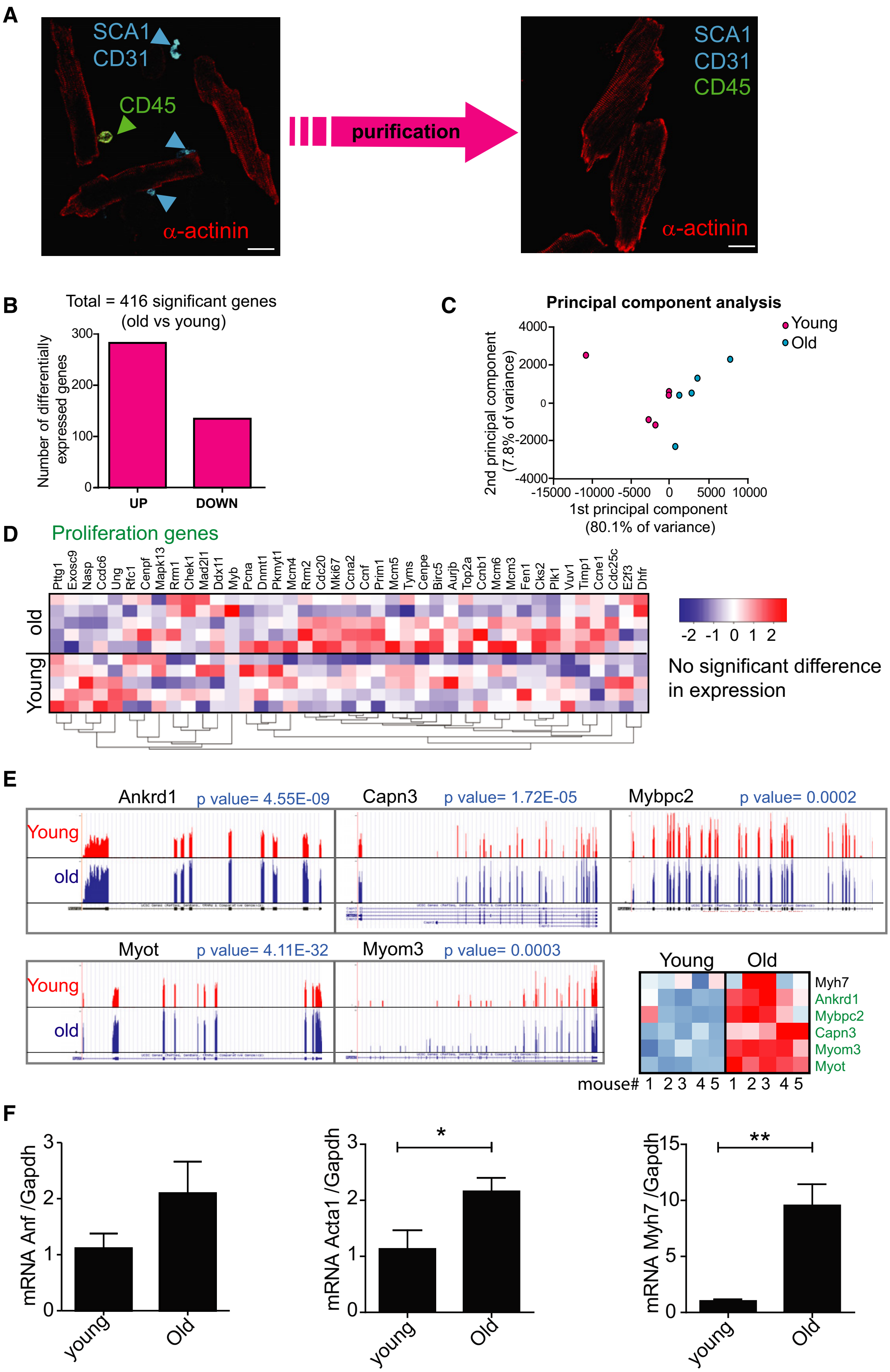
A
SCA1
CD31
CD45
α-actinin
purification
SCA1
CD31
CD45
α-actinin
B
Total = 416 significant genes
(old vs young)
Number of differentially expressed genes
UP
DOWN
C
Principal component analysis
Young
Old
2nd principal component (7.8% of variance)
1st principal component (80.1% of variance)
D
Proliferation genes
old
Young
No significant difference in expression
E
Ankrd1 p value= 4.55E-09
Capn3 p value= 1.72E-05
Mybpc2 p value= 0.0002
Myot p value= 4.11E-32
Myom3 p value= 0.0003
Young
old
Myh7
Ankrd1
Mybpc2
Capn3
Myom3
Myot
mouse# 1 2 3 4 5 1 2 3 4 5
F
mRNA Anf /Gapdh
mRNA Acta1 /Gapdh
mRNA Myh7 /Gapdh
young
Old

Figure EV3.

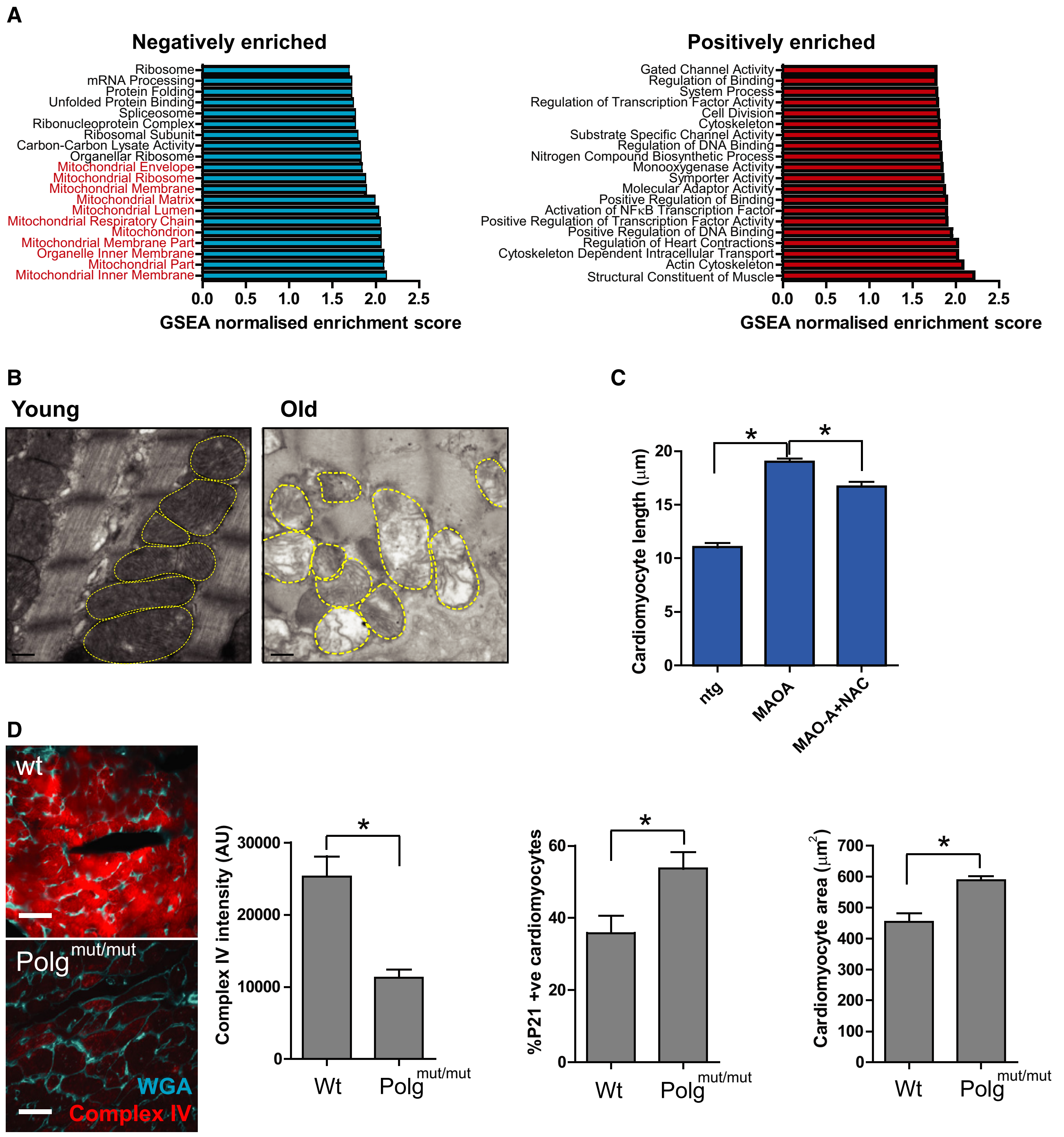

Figure EV4. Mitochondrial dysfunction induces senescence and hypertrophy in cardiomyocytes.

A Normalised GSEA enrichment scores for the top 20 positively and negatively enriched GO ontologies. Those negatively enriched ontologies associated with mitochondrial structure and function are highlighted in red.

B Transmission electron microscopy to detect mitochondrial ultrastructural defects in young (3 months) and old (20 months) mice. Scale bars represent 500 nm.

C Cardiomyocyte length analysis on WT, MAO-A or MAO-A mice with or without drinking water supplemented with 1.5 g/kg/day NAC from the age of 4–24 weeks. Data are mean ± SEM of n = 5–10 mice per group.

D Representative images of mitochondrial complex IV (red) and WGA (blue) in 12-month-old WT and Polg$^{mut/mut}$ mice. Graphs represent complex IV intensity (left), p21 positivity by immunohistochemistry and mean cardiomyocyte area (right). Data are mean ± SEM of n = 3–5 mice per group. More than 100 cardiomyocytes were quantified per group. Scale bar represents 20 µm.

Data information: Asterisks denote a statistical significance at $P < 0.05$ using two-tailed t-test.

© 2019 The Authors

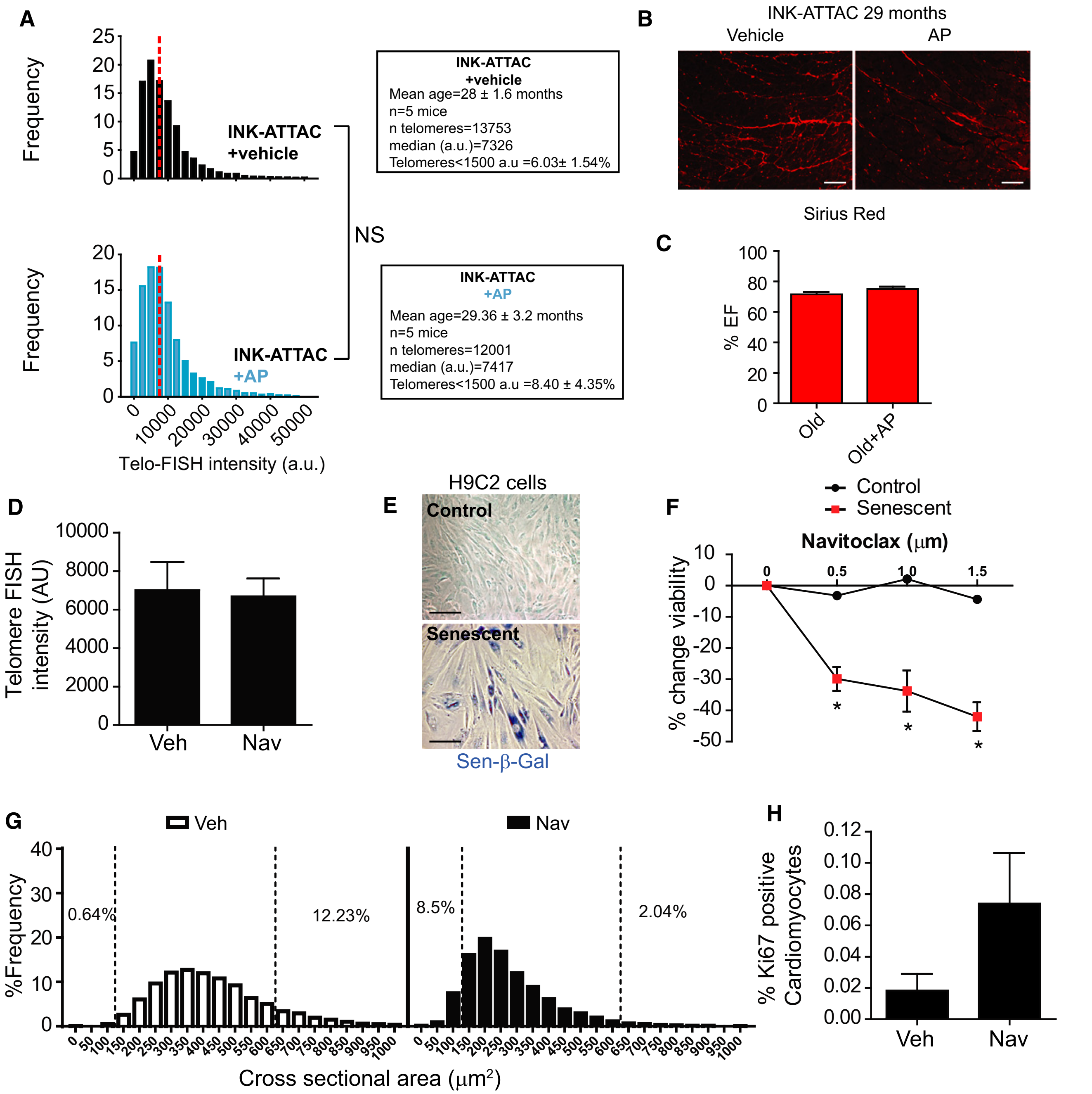

Figure EV5. Clearance of senescent cells does not impact on cardiomyocyte telomere length or cardiac function (% EF) but reduces fibrosis and hypertrophy.

A Histograms showing distribution of individual telomere intensities measured by Q-FISH comparing TAF and non-TAF in INK-ATTAC mice (28–29 months old) treated with vehicle or AP20187. > 150 cardiomyocytes were analysed per mouse.

B Representative image of Sirius Red staining in old INK-ATTAC mice treated AP20187 (or vehicle) at 29 months of age. Scale bar represents 50 μm.

C Ejection fraction in old INK-ATTAC treated with or without AP. Data are mean ± SEM of 11–12 mice per group.

D Mean telomere FISH intensity in 23-month-old mice with or without navitoclax treatment. Data are mean ± SEM of n = 3 per treatment group.

E H9C2 cardiomyoblasts were induced to senescence (representative Sen-β-Gal image) via exposure to 10 Gy X-ray radiation. Scale bar represents 50 μm.

F Navitoclax treatment significantly reduced cell viability in senescent cardiomyoblasts in a dose-dependent manner. Navitoclax had no effect on the viability of non-senescent cardiomyoblasts. Data are mean ± SEM n = 3 for each treatment group and dosage. Statistical analysis via two-way ANOVA; $^*P < 0.05$.

G Distribution of cardiomyocyte cross-sectional area in 23-month-old mice treated or not with navitoclax. Data show that most cardiomyocytes with areas above 650 μm^2 are not detected following navitoclax and smaller cardiomyocytes with areas below 100 μm^2 emerge. Seven animals per group were analysed.

H Quantification of Ki-67 positive cardiomyocytes in vehicle- and navitoclax-treated animals. Data are mean ± SEM of n = 3 per treatment group.

© 2019 The Authors
