# Supplementary material for: Length‐independent telomere damage drives post‐mitotic cardiomyocyte senescence
Source: EMBO J. 2019 Feb 8;38(5):e100492. doi: 10.15252/embj.2018100492 (PMC6396144; doi:10.15252/embj.2018100492)
Supplement: Supplementary file 3 — Movie EV1 [file EMBJ-38-e100492-s003.zip › MovieEV1_Legend.docx]

**Movie Expanded View 1.** Movie showing comparison of 3D reconstruction of Immuno-FISH using STED or confocal microscopy in CMs of 30 month old mice. Light blue STED – telo-FISH, red – confocal telo-FISH, green – γH2AX, dark blue DAPI.
