# Supplementary material for: Length‐independent telomere damage drives post‐mitotic cardiomyocyte senescence
Source: EMBO J. 2019 Feb 8;38(5):e100492. doi: 10.15252/embj.2018100492 (PMC6396144; doi:10.15252/embj.2018100492)
Supplement: Supplementary file 5 — Movie EV3 [file EMBJ-38-e100492-s005.zip › MovieEV3_Legend.docx]

**Movie Expanded View 3.** Movie of individual Z-stacks from confocal microscopy showing Edu incorporation in a mononucleated CM, post navitoclax treatment. Green-Troponin C, Red-Edu, White WGA and Blue-DAPI
